# Association of COVID-19 Lockdown With the Tumor Burden in Patients With Newly Diagnosed Metastatic Colorectal Cancer

**DOI:** 10.1001/jamanetworkopen.2021.24483

**Published:** 2021-09-08

**Authors:** Alain R. Thierry, Brice Pastor, Ekaterina Pisareva, Francois Ghiringhelli, Olivier Bouché, Christelle De La Fouchardière, Julie Vanbockstael, Denis Smith, Eric François, Mélanie Dos Santos, Damien Botsen, Stephen Ellis, Marianne Fonck, Thierry André, Emmanuel Guardiola, Faiza Khemissa, Benjamin Linot, J. Martin-Babau, Yves Rinaldi, Eric Assenat, Lea Clavel, Sophie Dominguez, Celine Gavoille, David Sefrioui, Veronica Pezzella, Caroline Mollevi, Marc Ychou, Thibault Mazard

**Affiliations:** 1 Institut de Recherche en Cancérologie de Montpellier (IRCM), Institut National de la Santé et de la Recherche Médicale (INSERM) U1194, Université de Montpellier, Institut Régional du Cancer de Montpellier, Montpellier, France; 2 Centre Georges François Leclerc, Dijon, France; 3 Hôpital Robert Debré, Reims, France; 4 Centre Léon Bérard, Lyon, France; 5 Institut de Cancérologie de l'Ouest, Angers, Saint-Herblain, France; 6 Hôpital Haut-Lévêque, Centre Hospitalier Universitaire (CHU) de Bordeaux, Pessac, France; 7 Centre Antoine Lacassagne, Nice, France; 8 Centre François Baclesse, Caen, France; 9 Medical Oncology Department, Godinot Institute, Reims, France; 10 Centre Catalan d'Oncologie, Perpignan, France; 11 Institut Bergonié, Bordeaux, France; 12 Hȏpital Saint-Antoine, Paris, France; 13 Centre de Cancérologie du grand Montpellier, Montpellier, France; 14 Centre Hospitalier de Perpignan, Perpignan, France; 15 Hôpital Privé du Confluent, Nantes, France; 16 Centre Cario, Plerin, France; 17 Hôpital Européen de Marseille, Marseille, France; 18 Department of Medical Oncology, St Eloi University Hospital, Montpellier, France; 19 Hôpital Privé Jean Mermoz, Lyon, France; 20 Hôpital Saint Vincent de Paul, Lille, France; 21 Institue de Cancérologie de Lorraine, Vadoeuvre-les-Nancy, France; 22 CHU de Rouen, Rouen, France; 23 Unicancer, Paris, France

## Abstract

**Importance:**

The COVID-19 pandemic has been associated with substantial reduction in screening, case identification, and hospital referrals among patients with cancer. However, no study has quantitatively examined the implications of this correlation for cancer patient management.

**Objective:**

To evaluate the association of the COVID-19 pandemic lockdown with the tumor burden of patients who were diagnosed with metastatic colorectal cancer (mCRC) before vs after lockdown.

**Design, Setting, and Participants:**

This cohort study analyzed participants in the screening procedure of the PANIRINOX (Phase II Randomized Study Comparing FOLFIRINOX + Panitumumab vs FOLFOX + Panitumumab in Metastatic Colorectal Cancer Patients Stratified by *RAS* Status from Circulating DNA Analysis) phase 2 randomized clinical trial. These newly diagnosed patients received care at 1 of 18 different clinical centers in France and were recruited before or after the lockdown was enacted in France in the spring of 2020. Patients underwent a blood-sampling screening procedure to identify their *RAS* and *BRAF* tumor status.

**Exposures:**

mCRC.

**Main Outcomes and Measures:**

Circulating tumor DNA (ctDNA) analysis was used to identify *RAS* and *BRAF* status. Tumor burden was evaluated by the total plasma ctDNA concentration. The median ctDNA concentration was compared in patients who underwent screening before (November 11, 2019, to March 9, 2020) vs after (May 14 to September 3, 2020) lockdown and in patients who were included from the start of the PANIRINOX study.

**Results:**

A total of 80 patients were included, of whom 40 underwent screening before and 40 others underwent screening after the first COVID-19 lockdown in France. These patients included 48 men (60.0%) and 32 women (40.0%) and had a median (range) age of 62 (37-77) years. The median ctDNA concentration was statistically higher in patients who were newly diagnosed after lockdown compared with those who were diagnosed before lockdown (119.2 ng/mL vs 17.3 ng/mL; *P* < .001). Patients with mCRC and high ctDNA concentration had lower median survival compared with those with lower concentration (14.7 [95% CI, 8.8-18.0] months vs 20.0 [95% CI, 14.1-32.0] months). This finding points to the potential adverse consequences of the COVID-19 pandemic and related lockdown.

**Conclusions and Relevance:**

This cohort study found that tumor burden differed between patients who received an mCRC diagnosis before vs after the first COVID-19 lockdown in France. The findings of this study suggest that CRC is a major area for intervention to minimize pandemic-associated delays in screening, diagnosis, and treatment.

## Key Points

**Question:**

What is the implication of the COVID-19 lockdown for the tumor burden of patients with a newly diagnosed metastatic colorectal cancer?

**Findings:**

In this cohort study of 80 patients with metastatic colorectal cancer, the tumor burden, which was evaluated using the circulating tumor DNA in plasma, appeared to be significantly higher in patients who received a diagnosis after lockdown compared with those who were diagnosed before lockdown (119.2 ng/mL vs 17.3 ng/mL). Patients with greater tumor burden had lower median survival than those with lower tumor burden.

**Meaning:**

In this study, the tumor burden of colorectal cancer varied and appeared to be associated with poor survival for those who received a postlockdown diagnosis, suggesting that this cancer is a major area for intervention to minimize COVID-19–associated diagnostic delay.

## Teaser

This cohort study assesses the association of local and national COVID-19 restrictions with tumor burden, as measured by total plasma circulating tumor DNA concentration, among patients newly diagnosed with metastatic colorectal cancer.

## Introduction

The unprecedented burden placed on health systems worldwide by the COVID-19 crisis has had numerous and substantial implications for cancer care.^1,2^ People have been more reluctant to come to health care facilities for services because of fear of infection, particularly those with cancer, given that cancer is considered a comorbidity. Reduction or suspension of screening programs and diagnostic services has been a factor in delays in diagnosis in many countries.^1,3,4,5^ Access to treatment has been restricted to minimize the risk of SARS-CoV-2 exposure during therapy procedures for patients with cancer.^3^ The reprioritization of human resources and equipment to COVID-19 pandemic management has also been associated with the provision of suboptimal or delayed care.^1,6^

These implications have been exacerbated by the COVID-19 containment measures implemented by different countries, which have tended to evolve from recommendations and restrictions to lockdowns at both the local and national levels.^1,2^ Such measures were initially seen in the first few months of 2020 in Asia and Oceania and had spread to Europe and North and South America by March, depending mainly on the date of the first SARS-CoV-2 infection cases in those areas.^2^ The sheer number of patients with COVID-19 infection necessitating hospitalization and critical care has continued to strain health services and already limited resources. Individual fears of contracting the virus as well as restrictions on movement imposed by local and national authorities have generated additional physical and psychological barriers for patients who need to access essential care.

We conducted a cohort study to evaluate the association of the COVID-19 pandemic lockdown with the tumor burden of patients who were newly diagnosed with metastatic colorectal cancer (mCRC) before vs after lockdown. To our knowledge, no such clinical evaluation has been performed thus far. Conventional circulating biomarkers, such as carcinoembryonic antigen (CEA) and carbohydrate antigen 19-9, do not fully satisfy the clinical requirements for monitoring colorectal cancer (CRC) tumor burden in clinical practice because of their moderate levels of sensitivity and specificity.^7^ Therefore, we used circulating tumor DNA (ctDNA) analysis to assess the patients’ tumor burden.

Circulating tumor DNA is a newly identified source of biological information that has attracted the attention of researchers and clinicians in numerous fields.^8^ It has substantial clinical potential in oncology, including in molecular profiling, detection of residual disease, control of treatment efficacy, detection of clonal resistance, surveillance of recurrence, and screening.^9^ It first showed its promise by contributing to companion tests as a liquid biopsy, and then it obtained European Medicines Agency approval for use in the detection of sensitizing and/or resistant somatic alterations in oncodrivers, such as those in lung cancer and melanoma, as a tool to guide clinicians in selecting targeted therapies.^9,10^ Numerous studies have reported that tumors secrete DNA into the bloodstream in quantities that are proportional to their masses,^11,12,13^ especially in the case of mCRC, according to several investigations^11,14,15,16^ and work that illustrated the association of total ctDNA concentration with increasing hepatic tumor mass as identified by magnetic resonance imaging (eFigure 1 in the Supplement). Thus, ctDNA offers analytical and clinical advantages over conventional antigenic biomarkers, such as CEA, and may be considered as a surrogate marker of disease progression, at least in mCRC.^14,15,17,18,19,20^

## Methods

This cohort study included patients from the screening procedure of the ongoing PANIRINOX study (Phase II Randomized Study Comparing FOLFIRINOX + Panitumumab vs FOLFOX + Panitumumab in Metastatic Colorectal Cancer Patients Stratified by *RAS* Status from Circulating DNA Analysis), who were recruited before and after the first COVID-19 lockdown was enacted in France in the spring of 2020. In the PANIRINOX trial, treatment (FOLFIRINOX [leucovorin, fluorouracil, irinotecan, and oxaliplatin] + panitumumab or mFOLFOX6 [modified fluorouracil, leucovorin, and oxaliplatin] + panitumumab) is allocated according to a randomization procedure. However, the present work was carried out on an ad hoc basis at the time of the screening procedure and before randomization. The PANIRINOX study was reviewed and approved by the human investigations committee Sud Méditerranée IV. All patients provided written informed consent before the screening procedure. This cohort study, along with other trial-related documents, received approval from Unicancer, the sponsor of the PANIRINOX study, which received authorization from the Agence Nationale de Sécurité du Médicament et des Produits de Santé and the Comités de Protection des Personnes, according to French national regulatory requirements. We followed the Strengthening the Reporting of Observational Studies in Epidemiology (STROBE) reporting guideline.^21^

The PANIRINOX study is a first-line, phase 2 randomized clinical trial that assesses the activity of a combination chemotherapy with fluorouracil, leucovorin, oxaliplatin, and panitumumab with or without irinotecan (FOLFOX + panitumumab vs FOLFIRINOX + panitumumab) in patients with unresectable mCRC, who were selected by their *RAS* (GenBank 6237) and *BRAF* (GenBank 673) tumor status, which was obtained from ctDNA analysis. To our knowledge, it is the first interventional study to use ctDNA as a companion test for selecting patients with mCRC for anti–estimated glomerular filtration rate targeted therapy (eAppendix in the Supplement). It involves 31 hospitals and cancer centers in France. Its primary end point is the complete response rate defined as the complete disappearance of metastatic lesions and CEA level normalization after a maximum of 12 treatment cycles. Among the major patient selection criteria are age 18 to 75 years, Eastern Cooperative Oncology Group Performance Status score of 0 or 1, no previous treatment for metastatic disease, and no previous use of oxaliplatin in an adjuvant setting (eAppendix in the Supplement).

In France, the first mandatory home lockdown of 2020 lasted 55 days, from March 17 to May 11. The PANIRINOX study screening was consequently interrupted for 53 days, starting on March 19 and ending on May 11. We compared the ctDNA concentration in all patients who underwent screening after the lockdown (from May 14, 2020, to September 3, 2020, a 110-day period) with the ctDNA concentration in all patients who underwent screening before the lockdown (from November 11, 2019, to March 9, 2020). These patients were newly diagnosed with mCRC and received care at 1 of 18 different clinical centers in France. We also compared the ctDNA concentration in the prelockdown and postlockdown groups and the fractional cohorts of those who were included from the start of the PANIRINOX study (June-September 2017, September 2017-January 2018, January-April 2018, April-August 2018, August-December 2018, December 2018-March 2019, March-July 2019, and July-November 2019). Preanalytical conditions of the ctDNA analysis followed strict guidelines and methodologies that have been previously validated.^22,23,24,25^

Patients were screened through a blood-sampling procedure to identify their *RAS* and *BRAF* tumor status according to plasma analysis of circulating cell-free DNA, using IntPlex technology (DiaDx SAS).^22,23^ Those whose tumors were considered as *RAS* and *BRAF* wild type were subsequently included in the PANIRINOX study if they fulfilled all other inclusion criteria (eAppendix in the Supplement). The present study, therefore, benefited from the accuracy with which ctDNA can evaluate tumor burden and from the trial’s rigorous inclusion procedure and reporting, all of which supported the accuracy of assessment needed to achieve the objective of this study.

We examined all patients who underwent screening before and after lockdown (N = 268), regardless of their *RAS* and *BRAF* sequence variation status, to preclude any potential bias associated with sequence variation status. Given the interventional impact of ctDNA analysis in the PANIRINOX study, the analysis was completed within 5 days of receipt of the blood samples. In addition to ctDNA parameters analysis, we simultaneously collected demographic and clinicobiological parameters that are known to have prognostic value in this setting.^26,27^

### Statistical Analysis

Statistical analysis of prelockdown and postlockdown data was performed with the GraphPad Prism, version 6.01 (GraphPad Software Inc) and survival analysis was conducted with Stata, version 16.0 (StataCorp LLC). Where appropriate, data were log transformed before statistical analysis. Continuous variables were compared using the Mann-Whitney test, and categorical variables were compared using the Pearson χ^2^ test. Median follow-up was calculated with the reverse Kaplan-Meier method. Overall survival, defined as the time between the date of first metastatic diagnosis and the date of death from any cause, was estimated with the Kaplan-Meier method and compared using the log-rank test. Correlation analysis was performed using the Spearman test. Hazard ratios (HRs) are given with their 95% CIs. A 2-sided *P* < .05 was considered to be statistically significant.

## Results

We analyzed the ctDNA concentration in 80 patients who underwent screening before (n = 40) or after (n = 40) the first COVID-19 lockdown in France in 2020. These patients included 48 men (60.0%) and 32 women (40.0%) and had a median (range) age of 62 (37-77) years.

**Figure 1.**
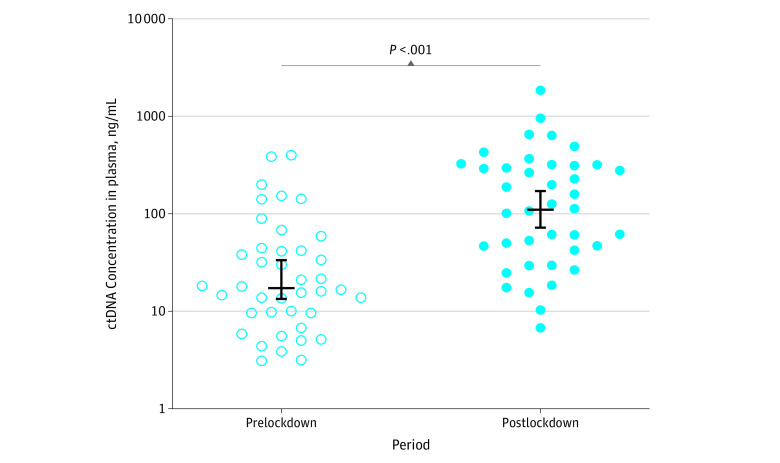
Comparison of Circulating Tumor DNA (ctDNA) Concentration in Patients With Newly Diagnosed Metastatic Colorectal Cancer in the Prelockdown and Postlockdown Periods The long horizontal bars indicate the median; shorter bars, the 95% CIs; and each dot, the ctDNA concentration in a single patient. The Mann-Whitney test was performed to compare the patient distributions and revealed a significant difference between the prelockdown and postlockdown periods.

**Figure 2.**
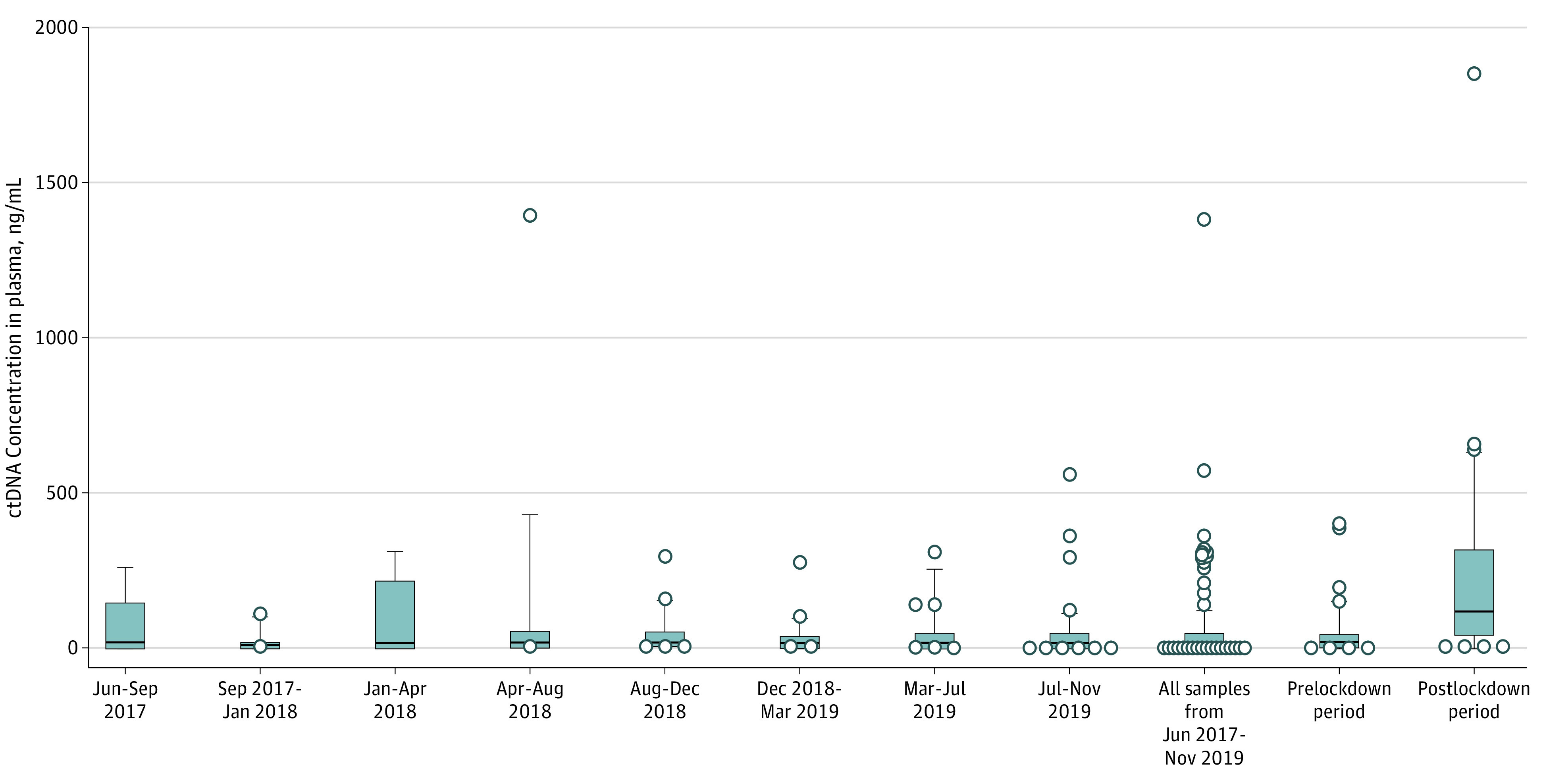
Comparison of Patients at the Start of the PANIRINOX Study and the Prelockdown Period The box plot represents circulating tumor DNA (ctDNA) concentration in patients in the 110-day fractional cohorts vs patients in the prelockdown (n = 40) and postlockdown (n = 40) periods. The Mann-Whitney test was performed to compare patient distributions. The horizontal bars inside the boxes indicate the median; error bars, the 10th to 90th percentile; squares, the median between the 25% percentile and the 75% percentile; whiskers, the 10th to 90th percentile; and each dot, the ctDNA concentration of a single patient outside the 10th to 90th percentile.

As shown in Figure 1, the median (interquartile range [IQR]) ctDNA concentration was 17.3 (9.57-43.78) ng/mL before lockdown and 119.2 (43.38-315.8) ng/mL after lockdown (eTable 1 in the Supplement). This postlockdown ctDNA concentration represented a 6.9-fold increase. A statistically significant difference between the 2 cohorts was observed (17.3 [95% CI, 13.58-33.52] vs 119.2 [95% CI, 53.13-278.1]; *P* < .001) (Figure 1 and Figure 2). The values obtained from patients included before lockdown (n = 40) were similar to those obtained from all patients in the fractional cohorts (n = 188), who were included in the PANIRINOX study starting 30 months before lockdown, showing a median (IQR) ctDNA concentration in plasma of 13.0 (6.43-46.13) ng/mL (Figure 2). In addition, the median (IQR) ctDNA concentration in the fractional cohorts showed no statistical difference from the levels in the prelockdown cohort (June-September 2017: 29.94 [5.27-149.2] ng/mL, *P* > .99; September 2017-January 2018: 9.13 [6.37-13.61] ng/mL, *P* = .07; January-April 2018: 18.36 [3-220.2] ng/mL, *P* = .71; April-August 2018: 18.51 [6.99-55.06] ng/mL, *P* = .99; August-December 2018: 13.38 [9.17-55.85] ng/mL, *P* = .89; December 2018-March 2019: 9.19 [4.72-40.91] ng/mL, *P* = .27; March-July 2019: 18.38 [5.11-49.25] ng/mL, *P* = .54; July-November 2019: 12.91 [7.05-49] ng/mL, *P* = 40), whereas they were statistically different from the levels in the postlockdown cohort (Figure 2).

**Figure 3.**
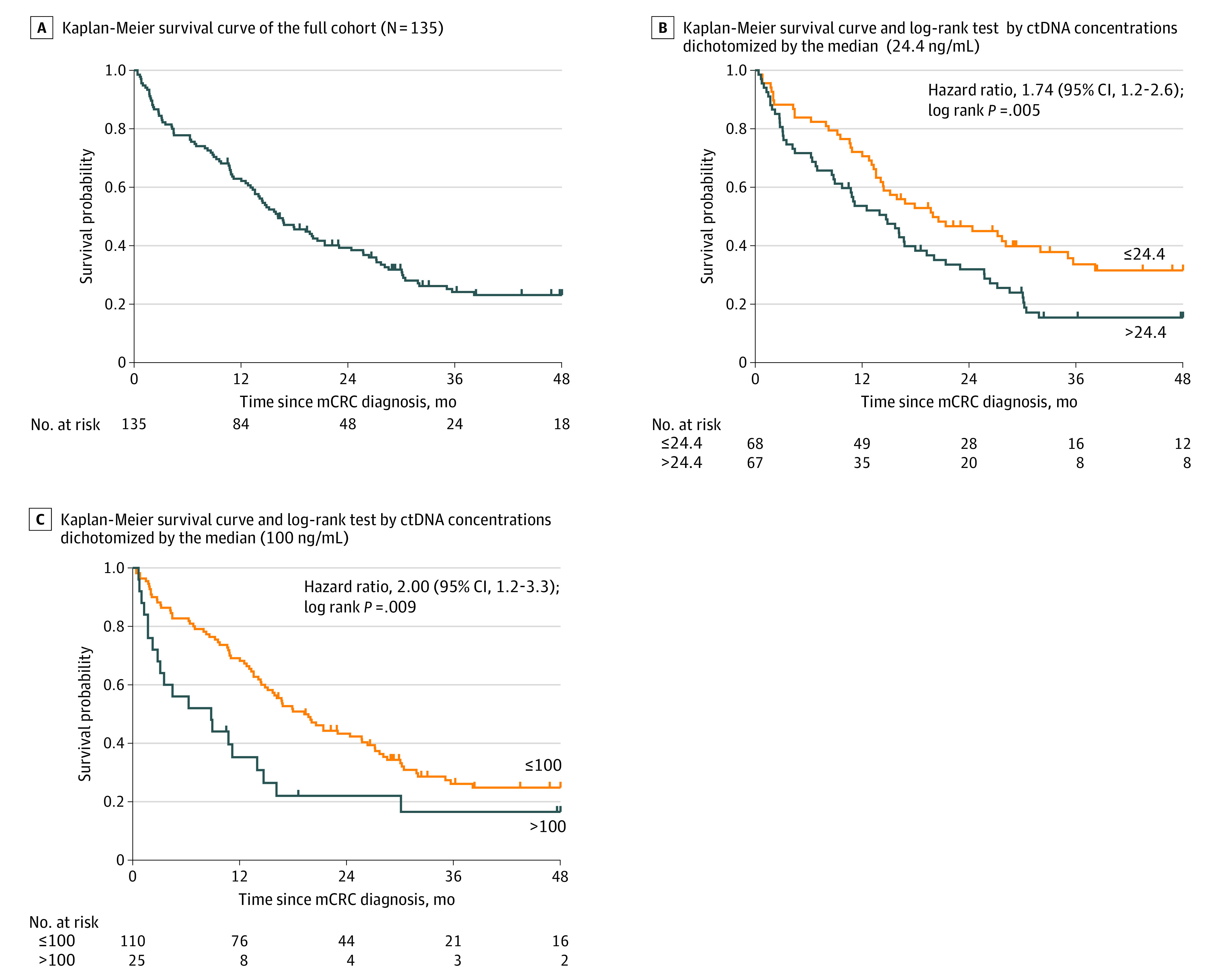
Overall Survival Analysis of Patients With Newly Diagnosed Metastatic Colorectal Cancer (mCRC) ctDNA indicates circulating tumor DNA.

Regarding patient characteristics, no difference was observed in the groups who received a diagnosis before vs after lockdown (Table; eFigures 2 and 3 in the Supplement). The delay of blood sample delivery was also similar, as was the alteration in ctDNA concentration and the alteration in allele frequency (eFigures 4 to 6; eTable 1 in the Supplement). For example, the median (IQR) alteration in allele frequency was 10.45% (0.88%-19.22%) in the prelockdown cohort and 6.18% (0.45%-21.96%) in the postlockdown cohort (eTable 1). The median white blood cell count, lactate dehydrogenase (LDH) level, and CEA level were slightly higher in the postlockdown vs prelockdown setting, but the differences were not statistically significant (Table; eFigures 7 to 9 in the Supplement). The ctDNA concentration was significantly associated with an increase in LDH level (*r* = 0.72; *P* < .001) and white blood cell count (*r* = 0.73; *P* < .001) in patients who underwent screening after lockdown. The CEA level was associated with an increase of ctDNA concentration in patients in the prelockdown (*r* = 0.38; *P* = .04) and postlockdown (*r* = 0.22; *P* = .24) groups (eFigures 10 to 14 in the Supplement). When dichotomizing this cohort by the median (IQR) ctDNA concentration (24.4 [2.3-1406] ng/mL), we found that patients who had higher ctDNA plasma concentration showed a statistically lower median survival (14.7 [95% CI, 8.8-18.0] months vs 20.0 [95% CI, 14.1-32.0] months; HR, 1.74 [95% CI, 1.2-2.6]; *P* = .005) (Figure 3B; eTable 2 in the Supplement).

**Table.** Patient Characteristics

Characteristic	No. (%)			*P* value
	Overall	Prelockdown group	Postlockdown group	
No. of patients	80 (100)	40 (50)	40 (50)	
Age, y				.86
Median (range)	62 (37-77)	63 (37-77)	61 (39-77)	
Missing data	1	0	1	
Sex				.65
Male	48 (60.0)	25 (62.5)	23 (57.5)	
Female	32 (40.0)	15 (37.5)	17 (42.5)	
Location of primary tumor				.84
Right colon	19 (24.0)	9 (23.1)	10 (25.0)	
Left colon	60 (76.0)	30 (76.9)	30 (75.0)	
Missing data	1	1	0	
Primary tumor in place				.81
Yes	57 (71.2)	28 (70.0)	29 (72.5)	
No	23 (28.8)	12 (30.0)	11 (27.5)	
No. of metastatic sites				
Median (range)	2 (1-4)	2 (1-3)	2 (1-4)	.30
1	28 (43.8)	15 (48.4)	13 (39.4)	.47
>1	36 (56.2)	16 (51.6)	20 (60.6)	
Missing data	16	9	7	
Liver involvement				.96
Yes	55 (84.6)	27 (84.4)	28 (84.9)	
No	10 (15.4)	5 (15.6)	5 (15.1)	
Missing data	15	8	7	
Limited liver disease				.81
Yes	23 (28.8)	12 (30.0)	11 (27.5)	
No	57 (71.2)	28 (70.0)	29 (72.5)	
LDH level, U/L				
Median (range)	345 (137-2690)	263 (148-2690)	410 (137-1256)	.46
<245	22 (39.3)	14 (48.3)	8 (29.6)	.18
≥245	34 (60.7)	15 (51.7)	19 (70.4)	
Missing data	24	11	13	
WBC count, G/L				
Median (range)	9.1 (4.4-27.3)	8.5 (4.8-22.4)	9.4 (4.4-27.3)	.31
<10	38 (62.3)	21 (67.7)	17 (56.7)	.37
≥10	23 (37.7)	10 (32.3)	13 (43.3)	
Missing data	19	9	10	
CEA level, ng/mL				
Median (range)	39.8 (0.7-13590)	34.0 (0.7-9902)	40.8 (1.4-13590)	.49
<5	9 (14.8)	4 (12.9)	5 (16.7)	.68
≥5	52 (85.2)	27 (87.1)	25 (83.3)	

Abbreviations: CEA, carcinoembryonic antigen; LDH, lactate dehydrogenase; WBC, white blood cell.

SI conversion factors: To convert CEA level to micrograms per liter, multiply by 1.0; LDH level to microkatal per liter, multiply by 0.0167; WBC count to ×10^9^/L, multiply by 0.001.

## Discussion

The differences in tumor burden between patients who were diagnosed before vs after lockdown and the resulting risk of reduced survival point to the association between the pandemic-related lockdown and unfavorable consequences for patients with newly diagnosed mCRC, who may have delayed their first visit to an oncologist. The lower number of mCRC diagnoses during the beginning of the COVID-19 pandemic^1,3^ may be associated with patients’ reluctance to visit a physician or health care facility. A possible reason for this reluctance was fear of COVID-19 infection or burdening the health system, as described by a quote from a patient with cancer^28^ (eAppendix in the Supplement). In addition to patients’ subjective anxieties and reticence, numerous reports observed the considerable delays in sending out millions of solicitations for bowel cancer screening and a backlog (in England alone) of thousands of individuals awaiting further investigation after receiving a positive screening result.^2,3^

Although the COVID-19 lockdown was a necessity, it led to unintended consequences in the diagnosis of various cancers. The pandemic has affected all aspects of the cancer care pathway, especially the areas of screening, diagnosis, and surgical treatment.^4,29,30^ For instance, De Vincentiis et al^4^ reported that the number of cancer diagnoses in Italy decreased by 39% in the first 6 months of 2020 compared with the mean number recorded in 2018 and 2019. The highest decreases in diagnosis rates were observed in prostate cancer (75%), bladder cancer (66%), and CRC (62%), when the number of new or first metastatic malignant diagnoses during lockdown (weeks 11-20 of 2020) was compared with the number in the same period in the previous 2 years.^4^ Given that colonoscopy numbers are closely associated with initial CRC diagnoses, a 55% decrease in colon examinations was found between March and April 2020, as reported by Cancer Australia.^31^

In addition to the abrupt reduction (86%) in preventive CRC screenings in the US after the declaration of the COVID-19 national emergency (March 1, 2020), a 64% decrease (ie, 95 000) in the number of colonoscopies performed between March 15 and June 16, 2020, compared with previous years has been reported.^31^ Furthermore, after June 16, 2020, weekly volumes remained 36% lower than the pre–COVID-19 levels.^31^ Particularly relevant to the present study is the finding of an observational Taiwanese cancer registry study based on 39 000 newly identified CRC cases that increases in the risk of death were significantly associated with the delay between diagnosis and treatment; the results for an interval of 31 to 150 days were an HR of 1.51 (95% CI, 1.43-1.59) and an HR of 1.64 (95% CI, 1.54-1.76) for 151 days or more.^32^ The French ONCOCARE-COV study (Oncology Care Pathway's Modifications Impact During COVID-19 Pandemic) confirmed a reduction in CRC fecal immunochemical test screenings (−86%), CRC biomolecular somatic analyses (−59%), and the number of new patient files being discussed in multidisciplinary tumor board meetings (−39%) during the 3-month lockdown period in 2020 compared with the same trimester in 2019.^29^ On a broader level, the ONCOCARE-COV study revealed the decreases in screening (−86% to −100%), diagnosis (−39%), and surgical treatment (−30%).^29^

Several studies have generated model-based estimates of the clinical consequences of delaying the first visit of patients who have been newly diagnosed with cancer.^33,34,35^ In the UK, Sud et al^34^ found that even a modest delay of 3 to 6 months in surgery for cancer may mitigate 19% to 43% of the life-years gained by hospitalization. Lai et al^36^ estimated that approximately 18 000 excess cancer deaths over the next 12 months may be attributed to the COVID-19 crisis. In the US, in addition to the 1 million deaths from breast cancer that are expected to occur in the next decade, approximately 10 000 deaths have been estimated as the outcome of pandemic-related delays of less than 6 months in screening and cancer care.^37^

The health outcomes of COVID-19–associated lockdowns are particularly notable in oncology, and repeated or extended lockdowns may lead to decreased surveillance and advance care planning. To address this threat, regulatory institutions, such as the American Society of Clinical Oncology and the European Society for Medical Oncology, established recommendations and guidance for delivering care to patients with cancer during the pandemic and lockdowns.^1,2^ To minimize risks to patients with gastrointestinal malignant neoplasms, for instance, the American College of Surgeons, Society of Surgical Oncology, French digestive oncology intergroup guideline (Thésaurus National de Cancérologie Digestive), and the European Society for Medical Oncology set new priorities, such as prioritizing surgery for colon cancer involving imminent obstruction or for locally advanced rectal cancer. Similarly, new priorities concerning CRC management were set by the Colorectal Cancer Alliance, Thésaurus National de Cancérologie Digestive, National Comprehensive Cancer Network, European Society for Medical Oncology, and the City of Hope National Medical Center.^1^ Such recommendations were used to reclassify and reprioritize ongoing CRC care and management during the lockdown.

When CRC is diagnosed early, the treatment outcome is more favorable. In a large meta-analysis, Hanna et al^33^ reported that even a 4-week delay in treatment was associated with increased mortality for 7 cancers, particularly CRC (HR, 1.04; 95% CI, 0.95-1.13). This quantitative observation, although focused on a small sample of a specific type of patient with cancer, showed that delays in diagnosis would unnecessarily cost lives and life-years. This increase in ctDNA concentration after lockdown is striking and points to the levels of tumor burden at diagnosis, which have been associated with patient survival.^33,38,39^

To estimate the association between tumor burden and survival, we retrospectively analyzed data from 2 previous clinical studies that examined ctDNA concentration in the same way.^22,23^ Each of these studies used an identical, rigorous method to assess ctDNA before patients began first-line chemotherapy. All patients with newly diagnosed mCRC were identified from their data.^22,23^ In the present study, patients who were diagnosed with higher ctDNA plasma concentration had a statistically lower median survival compared with those with lower ctDNA concentration. Such comparisons illustrate and anticipate the lockdown’s unfavorable implications for patient survival. The full lockdown-related consequences for patient survival will be examined in a future 3-year survival study.

In response to the proliferation of the virus and its variants, many countries will likely implement further lockdowns. Thus, we believe that corrective action should be taken to minimize the clinical implications of delayed cancer diagnosis, including (1) reinforcing mass screening using the fecal occult blood test, (2) improving the communication strategy to avoid late patient diagnosis, and (3) providing adequate resources and creating robust plans to deal with backlogs in diagnosis and treatment. Patient triage could be performed by a quick assessment of tumor burden and testing of biomarkers with predictive and prognostic value (such as immunohistochemistry for mismatch repair proteins; sequence variation analysis for *KRAS* [GenBank 3845], *NRAS* [GenBank 4893], and *BRAF)*. For this purpose, we believe that ctDNA analysis that reveals qualitative (tumor molecular profiling) or quantitative information^9,14,22,23,40^ may be an ideal tool, as previously reported.^17,20,41^ The diagnostic power of ctDNA would be largely improved by using a multianalyte approach.^42,43^ Such a strategy would include both qualitative (such as genetic or epigenetic alterations) and quantitative (such as tissue or cell of origin or structural characteristics) markers. Artificial intelligence may also help achieve this goal as highlighted in a recent report.^43^

Despite the growing number of reports about the magnitude of the burden that the pandemic has placed on health systems worldwide, no study has yet evaluated the increased tumor burden of patients who received a postlockdown cancer diagnosis. To our knowledge, this study was the first to assess the association between COVID-19 restrictions and delayed treatment and diagnostic services for a specific cancer. The findings suggest that CRC can benefit from interventions to minimize the adverse clinical outcomes of pandemic-associated delays.

### Limitations

This study has some limitations. Although LDH level, white blood cell count, and to a lesser extent, CEA level were associated with an increase of ctDNA concentration, we could not provide tumor volume assessment by imaging in this study. Nonetheless, ctDNA concentration offers strong additional power to the routinely assessed serum markers. Although numerous studies found that lockdown was associated with delays in care and care seeking, we could not draw a direct association between our observation on tumor burden and the distinct delays in care for the newly diagnosed patients enrolled in the PANIRINOX study. It would be premature to evaluate the outcomes of the delays in screening, diagnosis, and treatment. This exploratory study instead offers a snapshot of a situation that continues to evolve.

## Conclusions

This cohort study pointed out the differences in tumor burden for patients who were diagnosed before vs after COVID-19 lockdown, including risk of reduced survival for those with postlockdown diagnoses. The findings of this study suggest that CRC is a major area for intervention to minimize the clinical implications of a pandemic-associated diagnostic delay.

## Notes

Supplement. **eAppendix.** Materials and Methods **eFigure 1.** Illustration of the Correlation Between the Tumor Burden and Total cirDNA Level in Three Metachronous mCRC Patients (One Site) With Increasing Hepatic Tumor Mass as Determined by MRI **eFigure 2.** Comparison of the Age of the Newly Diagnosed mCRC Patients From the Pre- and Post-Lockdown Study Cohorts (N=80) **eFigure 3.** Comparison of the Gender of the Newly Diagnosed mCRC Patients From the Pre- and Post-Lockdown Study Cohorts (N=80) **eFigure 4.** Comparison of the Delivery Delay of Blood Samples From the Newly Diagnosed mCRC Patients From the Pre- and Post-Lockdown Study Cohorts **eFigure 5.** Comparison of the Mutant cirDNA Concentration in the Newly Diagnosed Mutant mCRC Patients From the Pre- and Post-Lockdown Study Cohorts (N=48) **eFigure 6.** Comparison of the Mutant Allele Frequency in the Newly Diagnosed Mutant mCRC Patients From the Pre- and Post-Lockdown Study Cohorts (N=48) **eFigure 7.** Comparison of the Lactate Dehydrogenase (LDH) of the Newly Diagnosed mCRC Patients From the Pre- and Post-Lockdown Study Cohorts (N=56) **eFigure 8.** Comparison of the White Blood Cell Count in the Newly Diagnosed mCRC Patients From the Pre- and Post-Lockdown Study Cohorts (N=61) **eFigure 9.** Comparison of the Carcinoembryonic Antigen (CEA) in the Newly Diagnosed mCRC Patients From the Pre- and Post-Lockdown Study Cohorts (N=61) **eFigure 10.** Pearson r Correlation Analysis of the cirDNA, LDH, White Blood Cell Count, and CEA Levels in the Newly Diagnosed mCRC Patients From the Pre- and Post-Lockdown Study Cohorts **eFigure 11.** Scatter Plots Showing the Correlation Between LDH and cirDNA Concentrations in the Pre- and Post-Lockdown Cohorts **eFigure 12.** Scatter Plots Showing the Correlation Between White Blood Cell Count and cirDNA Concentrations in the Pre- and Post-Lockdown Cohorts **eFigure 13.** Scatter Plots Showing the Correlation Between CEA and cirDNA Concentrations in the Pre- and Post-Lockdown Cohorts **eFigure 14.** Scatter Plots Showing the Correlation Between White Blood Cell Count and LDH Concentration in the Pre- and Post-Lockdown Cohorts **eTable 1.** CirDNA Analysis for Pre-Lockdown and Post-Lockdown Cohorts **eTable 2.** Cox Models Data on Median Survival of mCRC Patients
