# Supplementary material for: Association of COVID-19 Lockdown With the Tumor Burden in Patients With Newly Diagnosed Metastatic Colorectal Cancer
Source: JAMA Netw Open. 2021 Sep 8;4(9):e2124483. doi: 10.1001/jamanetworkopen.2021.24483 (PMC8427376; doi:10.1001/jamanetworkopen.2021.24483)
Supplement: Supplement. — eAppendix. Materials and Methods eFigure 1. Illustration of the Correlation Between the Tumor Burden and Total cirDNA Level in Three Metachronous mCRC Patients (One Site) With Increasing Hepatic Tumor Mass as Determined by MRI eFigure 2. Comparison of the Age of the Newly Diagnosed mCRC Patients From the Pre- and Post-Lockdown Study Cohorts (N=80) eFigure 3. Comparison of the Gender of the Newly Diagnosed mCRC Patients From the Pre- and Post-Lockdown Study Cohorts (N=80) eFigure 4. Comparison of the Delivery Delay of Blood Samples From the Newly Diagnosed mCRC Patients From the Pre- and Post-Lockdown Study Cohorts eFigure 5. Comparison of the Mutant cirDNA Concentration in the Newly Diagnosed Mutant mCRC Patients From the Pre- and Post-Lockdown Study Cohorts (N=48) eFigure 6. Comparison of the Mutant Allele Frequency in the Newly Diagnosed Mutant mCRC Patients From the Pre- and Post-Lockdown Study Cohorts (N=48) eFigure 7. Comparison of the Lactate Dehydrogenase (LDH) of the Newly Diagnosed mCRC Patients From the Pre- and Post-Lockdown Study Cohorts (N=56) eFigure 8. Comparison of the White Blood Cell Count in the Newly Diagnosed mCRC Patients From the Pre- and Post-Lockdown Study Cohorts (N=61) eFigure 9. Comparison of the Carcinoembryonic Antigen (CEA) in the Newly Diagnosed mCRC Patients From the Pre- and Post-Lockdown Study Cohorts (N=61) eFigure 10. Pearson r Correlation Analysis of the cirDNA, LDH, White Blood Cell Count, and CEA Levels in the Newly Diagnosed mCRC Patients From the Pre- and Post-Lockdown Study Cohorts eFigure 11. Scatter Plots Showing the Correlation Between LDH and cirDNA Concentrations in the Pre- and Post-Lockdown Cohorts eFigure 12. Scatter Plots Showing the Correlation Between White Blood Cell Count and cirDNA Concentrations in the Pre- and Post-Lockdown Cohorts eFigure 13. Scatter Plots Showing the Correlation Between CEA and cirDNA Concentrations in the Pre- and Post-Lockdown Cohorts eFigure 14. Scatter Plots Showing the Correlation Bet [file jamanetwopen-e2124483-s001.pdf]

Supplementary Online Content

Thierry AR, Pastor B, Pisareva E, et al. Association of COVID-19 lockdown with the tumor burden in patients with newly diagnosed metastatic colorectal cancer. *JAMA Netw Open*. 2021;4(9):e2124483. doi:10.1001/jamanetworkopen.2021.24483

eAppendix. Materials and Methods

eFigure 1. Illustration of the Correlation Between the Tumor Burden and Total cirDNA Level in Three Metachronous mCRC Patients (One Site) With Increasing Hepatic Tumor Mass as Determined by MRI

eFigure 2. Comparison of the Age of the Newly Diagnosed mCRC Patients From the Pre- and Post-Lockdown Study Cohorts (N=80)

eFigure 3. Comparison of the Gender of the Newly Diagnosed mCRC Patients From the Pre- and Post-Lockdown Study Cohorts (N=80)

eFigure 4. Comparison of the Delivery Delay of Blood Samples From the Newly Diagnosed mCRC Patients From the Pre- and Post-Lockdown Study Cohorts

eFigure 5. Comparison of the Mutant cirDNA Concentration in the Newly Diagnosed Mutant mCRC Patients From the Pre- and Post-Lockdown Study Cohorts (N=48)

eFigure 6. Comparison of the Mutant Allele Frequency in the Newly Diagnosed Mutant mCRC Patients From the Pre- and Post-Lockdown Study Cohorts (N=48)

eFigure 7. Comparison of the Lactate Dehydrogenase (LDH) of the Newly Diagnosed mCRC Patients From the Pre- and Post-Lockdown Study Cohorts (N=56)

eFigure 8. Comparison of the White Blood Cell Count in the Newly Diagnosed mCRC Patients From the Pre- and Post-Lockdown Study Cohorts (N=61)

eFigure 9. Comparison of the Carcinoembryonic Antigen (CEA) in the Newly Diagnosed mCRC Patients From the Pre- and Post-Lockdown Study Cohorts (N=61)

eFigure 10. Pearson r Correlation Analysis of the cirDNA, LDH, White Blood Cell Count, and CEA Levels in the Newly Diagnosed mCRC Patients From the Pre- and Post-Lockdown Study Cohorts

eFigure 11. Scatter Plots Showing the Correlation Between LDH and cirDNA Concentrations in the Pre- and Post-Lockdown Cohorts

© 2021 Thierry AR et al. *JAMA Network Open.*

eFigure 12. Scatter Plots Showing the Correlation Between White Blood Cell Count and cirDNA Concentrations in the Pre- and Post-Lockdown Cohorts

eFigure 13. Scatter Plots Showing the Correlation Between CEA and cirDNA Concentrations in the Pre- and Post-Lockdown Cohorts

eFigure 14. Scatter Plots Showing the Correlation Between White Blood Cell Count and LDH Concentration in the Pre- and Post-Lockdown Cohorts

eTable 1. CirDNA Analysis for Pre-Lockdown and Post-Lockdown Cohorts

eTable 2. Cox Models Data on Median Survival of mCRC Patients

This supplementary material has been provided by the authors to give readers additional information about their work.

eAppendix. Materials and Methods

1. CirDNA analysis

All blood samples were collected in 10-milliter (mL) Streck tubes in all 18 clinical centers that sent by express mail the day or the day after blood draw following strict procedural guidelines. The IRCM U1094/ INSERM laboratory received blood samples within 5 days post-blood draw and centralized all following procedures towards cirDNA analysis under stringent pre-analytical guidelines reported by the laboratory[24]. The blood was then centrifuged at 1,200 g at 4°C for 10 minutes. The supernatants were isolated in sterile 1.5 mL Eppendorf tubes and centrifuged at 16,000 g at 4°C for 10 minutes. Afterwards, the plasma was either immediately used for DNA extraction or stored at -20°C. CirDNA was extracted from 1 mL of plasma using the QIAmp DNA Mini Blood kit (Qiagen) according to the ‘‘Blood and body fluid protocol.’’ DNA extracts were kept at -20°C until used[25].

The PANIRINOX clinical study implies the use of the IntPlex methodology to select patients showing WT mutational status for *KRAS*, *NRAS* and *BRAF* genes. IntPlex was established by our team and is based on an allele-specific blocker Q-PCR–based method specific for cirDNA analysis to enable the detection of point mutation and to determine mutant allele concentration[20]. This method combined the use of (1) allele-specific Q-PCR with blocking 3′-phosphate–modified oligonucleotide; (2) low Tm primers with mutation in 3′; (3) an integrated primer design; (4) routine internal positive and negative controls, and (5) optimal analytical procedures. This method enables the determination of the presence of a mutation, as well as the total concentration of cirDNA, the concentration of mutant cirDNA, the mutant allele frequency, and an index of DNA integrity. Note, the total cirDNA concentration value is determined by targeting a *KRAS* WT sequence, and is internally controlled by targeting a *BRAF* WT sequence for each sample[25].

CirDNA quantification methodology and the data description were carried out according to the MIQE guidelines[20]. Q-PCR amplifications were carried out at least in duplicate in a 25-µl reaction volume on CFX96 thermocycler (Bio-Rad) using the Bio-Rad CFX manager. Each PCR mixture was composed of 12.5 µl of PCR mix (Bio-Rad Supermix SYBR Green), 2.5 µl of each amplification primer (0.3 pmol/µl), 2.5 µl of PCR-analyzed water or 2.5 µl of oligoblocker, and 5 µl of DNA extract. Thermal cycling consisted of three repeated steps: a 3-minute hot-start polymerase activation-denaturation step at 95°C followed by 40 repeated cycles at 95°C for 10 seconds and then at 60°C for 30 seconds. Melting curves were obtained by increasing the temperature from 55 to 90°C with a plate reading every 0.2°C.
In the PANIRINOX study, 28 different mutations on the *KRAS*, *BRAF* and *NRAS* genes actionable in mCRC management care are tested. The IntPlex assay is clinically validated[20],[21], and shows the highest sensitivity and specificity so far described[8]. Poisson law experiments demonstrated that this method could accurately detect down to one molecule per PCR reaction mixture[21], with detection sensitivity as high as 1/100,000.

Intra- and inter-reproducibility experiments combining pre-analytic and analytic procedures demonstrated that the coefficient of variation for cirDNA concentration measurement is 24%[25]. IntPlex has been validated in two clinical studies[20],[21] is currently involved in 9 others, and has already enabled the testing of more than 2,000 individuals and 4,500 plama samples.

© 2021 Thierry AR et al. *JAMA Network Open.*

2. Ad hoc retrospective study

The increase seen in cirDNA values post-lockdown is striking, and points to levels of tumor burden at diagnosis which have been shown to affect patient survival [38,39]. To estimate the potential impact on survival of diagnostic delays arising from the lockdown, we retrospectively analyzed data accumulated during our last two clinical studies[21,22] from which we identified "all comers" newly diagnosed patients with mCRC , and in which a rigorously identical methodology was used to assess cirDNA (N=135) before patients began first line chemotherapy. The median cirDNA concentration (24.4 ng/mL, range [2.3-1406], Suppl. Table 2) of these patients is similar to that observed in the pre-lockdown cohort studied from the PANIRINOX trial. As observed in Figure 3A, patient with mCRC median survival in those two clinical studies (performed after a median follow up of 48.7 months, 95% CI [43.3-55.4]) is 16.2 months (N=135; 95% CI [13.6-20.6]). The median survival rate is low when compared with that seen in the latest literature. This may be attributed to the starting date of these two clinical studies (2009 and 2014), and to a less stringent patient selection compared to the PANIRINOX study; it nonetheless fits within the median survival range as previously observed in a meta-analysis30 (16-23 months). When dichotomizing this cohort by the cirDNA concentration median value (24.4 ng/mL, range [2.3-1406]), patients diagnosed with higher cirDNA plasma amounts showed a statistically lower median survival (14.7 (95% CI [8.8-18.0]) vs 20.0 (95% CI [14.1-32.0]) months, HR: 1.74, 95% CI [1.2-2.6]; P=0.005) (Figure 3B, Suppl. Table 2). When dichotomizing the cohort by 100 ng/mL, patients diagnosed with higher cirDNA plasma amounts also showed a statistically lower median survival (8.80, 95% CI [2.8-14.0] vs 19.3, 95% CI [2.8-14.0]) months; HR: 2.00, 95% CI [1.2-3.3] P=0.009) (Figure 3C, Suppl. Table 2). The comparative study of the lockdown impact (Figure 1) revealed 23 out of 40 patients (58%) showing a cirDNA amount over 100 ng/mL post-lockdown, and only 6 out of 40 patients (15%) above this concentration in pre-lockdown period. This could mean that 17 (23 minus 6) or 43% (58% minus 15%) supplementary patients diagnosed post-lockdown would have a median survival which is 54% (from 19.3 to 8.8 ng/mL) less than that of patients diagnosed pre- lockdown, presuming care management was equivalent, despite the difference in time period between survival cohort and study cohort. We are aware that this remains an assumption, but one which nonetheless illustrates and anticipates the lockdowns' marked deleterious impact on these patients' health.

3. Illustration of pandemic related anxiety by a cancer patient quote.

From a sixty year- Australian old patient, currently being treated for jaw cancer, who said: "*Because I didn't think it was an emergency, I didn't make any moves to go back to see anybody. And also because it was in the middle of COVID I was afraid of getting COVID or giving COVID, and so I just put up with it for a while*."[31]

4. Statistics

Statistical analysis of pre- versus post-lockdown data was performed using the GraphPad Prism V6.01 software and survival analysis with STATA 16.0 software. Where appropriate, data were log transformed prior to statistical analysis. Continuous variables were compared using the Mann-Whitney test. Categorical variables were compared using the Pearson's chi-square test. Median follow-up was calculated using the reverse Kaplan-Meier method. Overall survival (OS) was estimated using the Kaplan-Meier method, and compared using the Log-rank test. OS was defined as the time between the date of first metastatic diagnosis and the date of death from any cause. Hazard ratios (HR) are given with their 95% confidence interval (95% CI). Correlation analysis were performed using

© 2021 Thierry AR et al. *JAMA Network Open.*

the spearman test. A probability of less than 0.05 was considered to be statistically significant; *p<0.05, **p<0.01; ***p< 0.001; ****p< 0.0001.

eFigure 1. Illustration of the Correlation Between the Tumor Burden and Total cirDNA Level in Three Metachronous mCRC Patients (One Site) With Increasing Hepatic Tumor Mass as Determined by MRI

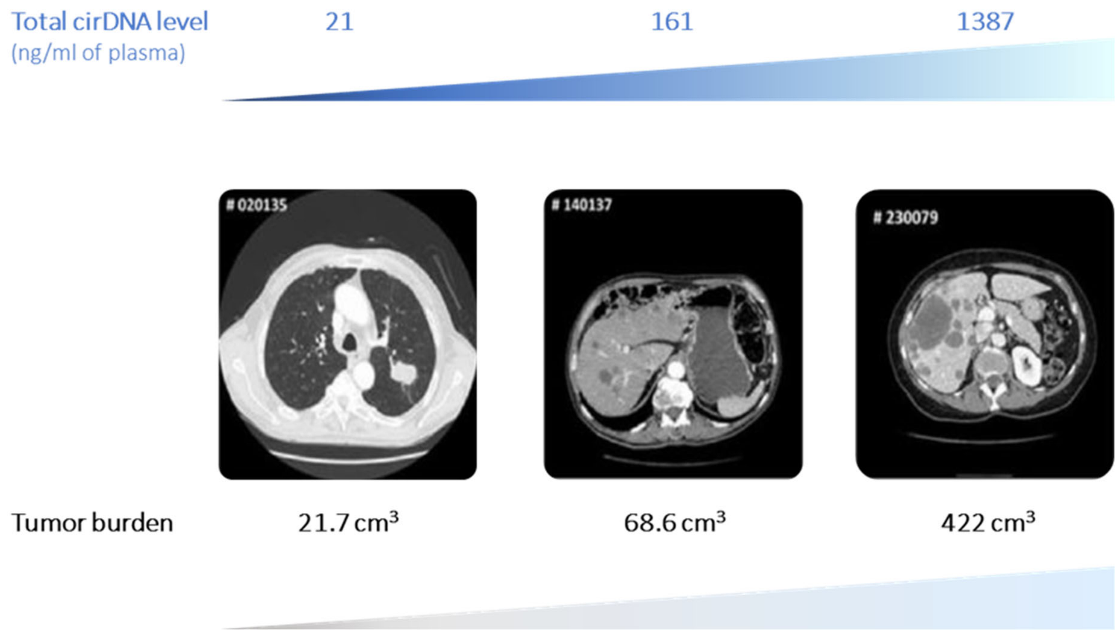

(Adapted from our previous report: El Messaoudi et al, Clinical Cancer Research, 2013, ref #16.)

Similar observation was reported by numerous other groups such as for:

mCRC (in addition to #10, 13-15 references) :

- Hamfjord, J.; Guren, T. K.; Dajani, O.; Johansen, J. S.; Glimelius, B.; Sorbye, H.; Pfeiffer, P.; Lingjærde, O. C.; Tveit, K. M.; Kure, E. H.; Pallisgaard, N.; Spindler, K.-L. G. Total Circulating Cell-Free DNA as a Prognostic Biomarker in Metastatic Colorectal Cancer before First-Line Oxaliplatin-Based Chemotherapy. Annals of Oncology 2019, 30 (7), 1088–1095. https://doi.org/10.1093/annonc/mdz139.
- Reece M, Saluja H, Hollington P, Karapetis CS, Vatandoust S, Young GP and Symonds EL. The Use of Circulating Tumor DNA to Monitor and Predict Response to Treatment in Colorectal Cancer. Front. Genet. 2019, 10:1118. doi:10.3389/fgene.2019.01118

other cancers (in addition to #10, 12, 13 references) :

- Nygaard AD, Holdgaard PC, Spindler K-LG, Pallisgaard N, Jakobsen A. The correlation between cell-free DNA and tumour burden was estimated by PET/CT in patients with advanced NSCLC. Br J Cancer 2014;110:363–8
- Tissot C, Toffart AC, Villar S, Souquet PJ, Merle P, Moro-Sibilot D, Pérol M, Zavadil J, Brambilla C, Olivier M, Couraud S. Circulating free DNA concentration is an

© 2021 Thierry AR et al. *JAMA Network Open.*

independent prognostic biomarker in lung cancer. Eur Respir J. 2015 Dec;46(6):1773-80. doi: 10.1183/13993003.00676-2015. Epub 2015 Oct 22. PMID: 26493785.

© 2021 Thierry AR et al. *JAMA Network Open.*

eFigure 2. Comparison of the Age of the Newly Diagnosed mCRC Patients From the Pre- and Post-Lockdown Study Cohorts (N=80)

AGE vs. cirDNA concentration

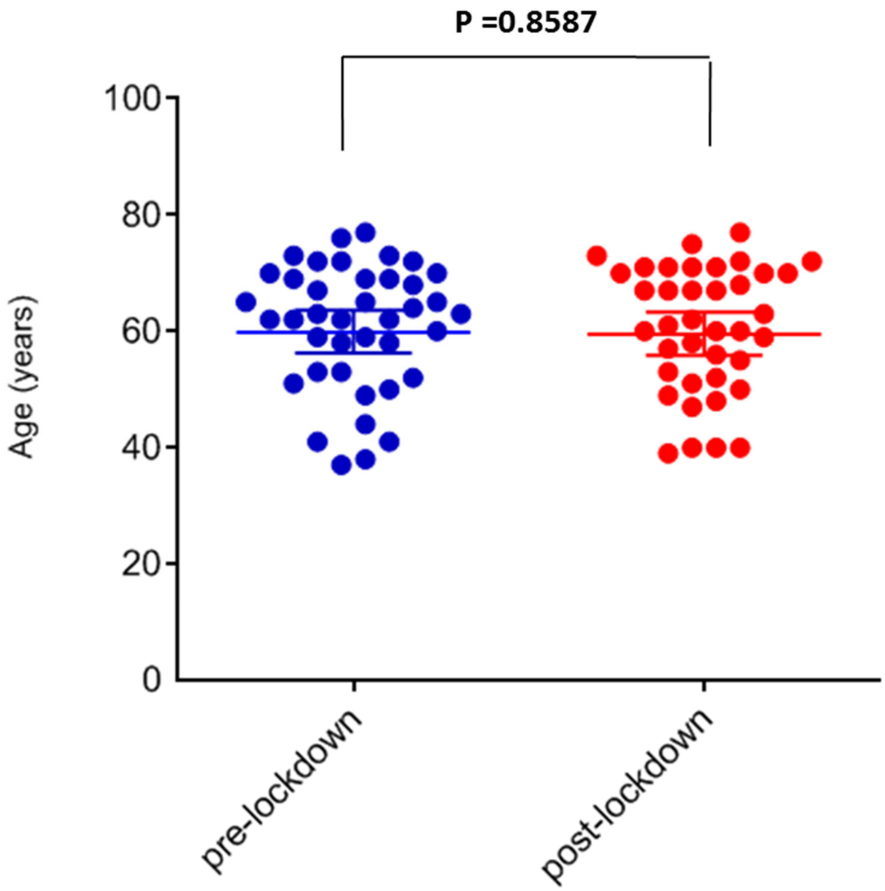

Group median is represented by a horizontal bold bar. Mann-Whitney U test was performed to compare distribution in pre- and post-lockdown patients. Each dot (blue, pre-lockdown; red, post-lockdown) represents the values of a single patient.

© 2021 Thierry AR et al. *JAMA Network Open.*

eFigure 3. Comparison of the Gender of the Newly Diagnosed mCRC Patients From the Pre- and Post-Lockdown Study Cohorts (N=80)

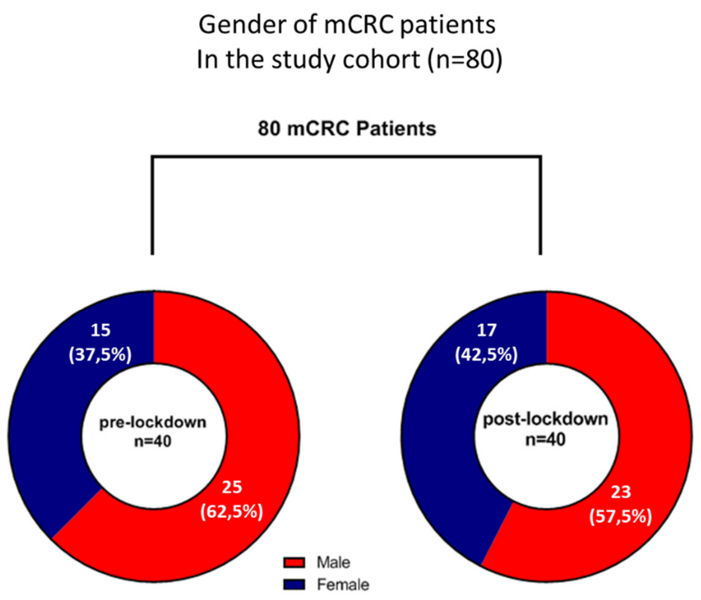

© 2021 Thierry AR et al. *JAMA Network Open.*

eFigure 4. Comparison of the Delivery Delay of Blood Samples From the Newly Diagnosed mCRC Patients From the Pre- and Post-Lockdown Study Cohorts

Delay of blood samples delivery (n=80)

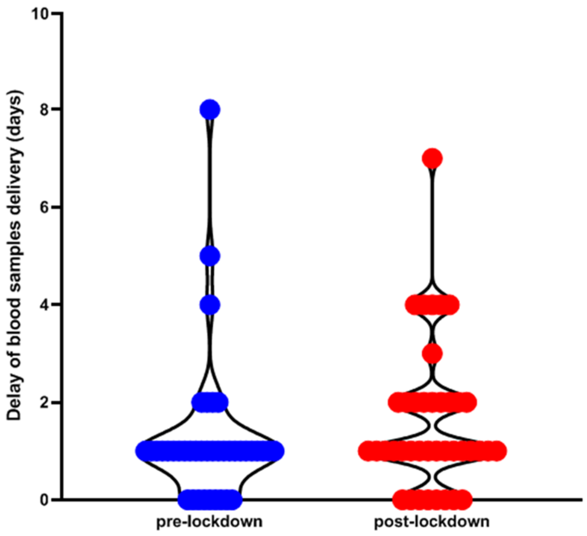

Table Analyzed	delay blood samples delivery
Column B	post-lockdown
vs.	vs,
Column A	pre-lockdown
Mann Whitney test	
P value	0,1198
Exact or approximate P value?	Exact
P value summary	ns
Significantly different (P < 0.05)?	No
One- or two-tailed P value?	Two-tailed
Sum of ranks in column A,B	1470 , 1771
Mann-Whitney U	649,5
Difference between medians	
Median of column A	1,000, n=40
Median of column B	1,000, n=40
Difference: Actual	0,000
Difference: Hodges-Lehmann	0,000

Mann-Whitney U test was performed to compare distribution in pre- and post-lockdown patients. Each dot (blue, pre-lockdown; red, post-lockdown) represents the values of a single patient.

© 2021 Thierry AR et al. *JAMA Network Open.*

eFigure 5. Comparison of the Mutant cirDNA Concentration in the Newly Diagnosed Mutant mCRC Patients From the Pre- and Post-Lockdown Study Cohorts (N=48)

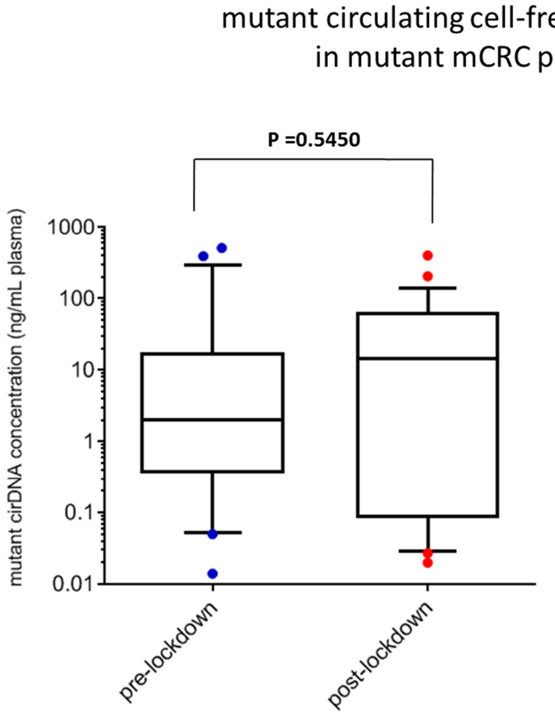

Table Analyzed	mutant cirDNA conc,
Column B	post-lockdown
vs.	vs,
Column A	pre-lockdown
Mann Whitney test	
P value	0,5450
Exact or approximate P value?	Exact
P value summary	ns
Significantly different (P < 0.05)?	No
One- or two-tailed P value?	Two-tailed
Sum of ranks in column A,B	509 , 667
Mann-Whitney U	256
Difference between medians	
Median of column A	1,985, n=22
Median of column B	14,21, n=26
Difference: Actual	12,23
Difference: Hodges-Lehmann	1,710

Group median is represented by a horizontal bold bar. Mann-Whitney U test was performed to compare distribution in pre- and post-lockdown patients. Each dot (blue, pre-lockdown; red, post-lockdown) represents the values of a single patient.

© 2021 Thierry AR et al. *JAMA Network Open.*

eFigure 6. Comparison of the Mutant Allele Frequency in the Newly Diagnosed Mutant mCRC Patients From the Pre- and Post-Lockdown Study Cohorts (N=48)

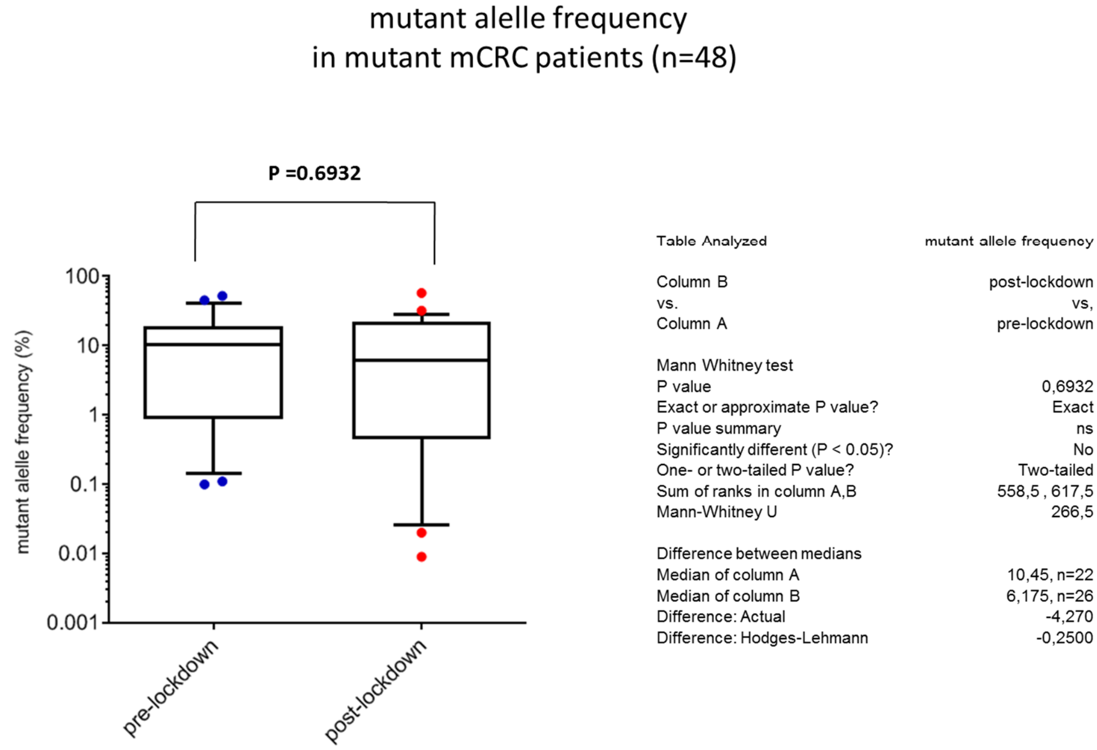

Table Analyzed	mutant allele frequency
Column B	post-lockdown
vs.	vs,
Column A	pre-lockdown
Mann Whitney test	
P value	0,6932
Exact or approximate P value?	Exact
P value summary	ns
Significantly different (P < 0.05)?	No
One- or two-tailed P value?	Two-tailed
Sum of ranks in column A,B	558,5 , 617,5
Mann-Whitney U	266,5
Difference between medians	
Median of column A	10,45, n=22
Median of column B	6,175, n=26
Difference: Actual	-4,270
Difference: Hodges-Lehmann	-0,2500

Group median is represented by a horizontal bold bar. Mann-Whitney U test was performed to compare distribution in pre- and post-lockdown patients. Each dot (blue, pre-lockdown; red, post-lockdown) represents the values of a single patient.

eFigure 7. Comparison of the Lactate Dehydrogenase (LDH) of the Newly Diagnosed mCRC Patients From the Pre- and Post-Lockdown Study Cohorts (N=56)

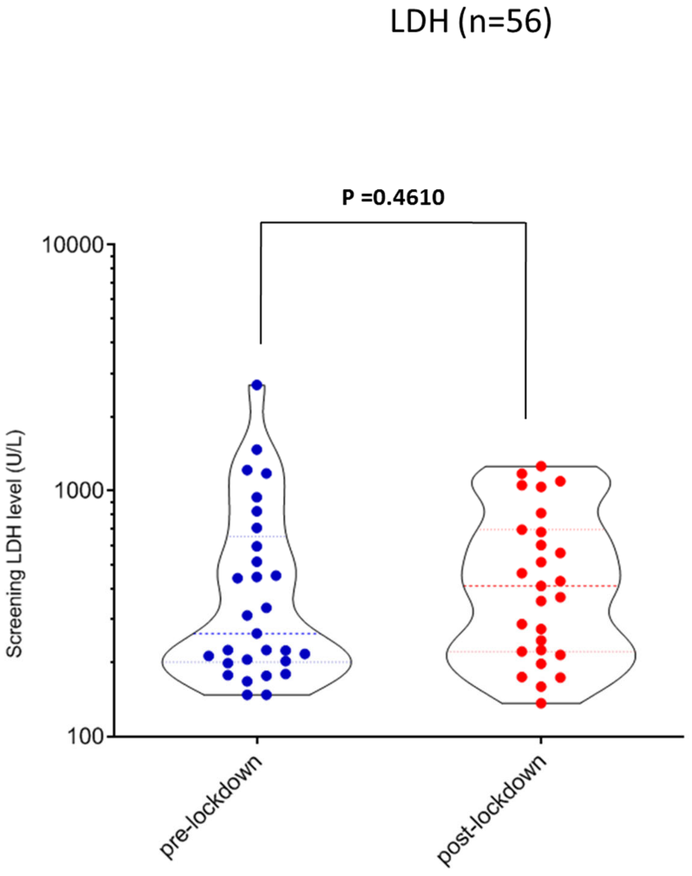

Table Analyzed	LDH Lockdown
Column B	pre-lockdown
vs.	vs,
Column A	post-lockdown
Mann Whitney test	
P value	0,4610
Exact or approximate P value?	Exact
P value summary	ns
Significantly different (P < 0.05)?	No
One- or two-tailed P value?	Two-tailed
Sum of ranks in column A,B	781 , 815
Mann-Whitney U	346
Difference between medians	
Median of column A	263,0, n=29
Median of column B	410,0, n=27
Difference: Actual	147,0
Difference: Hodges-Lehmann	42,00

Group median is represented by a horizontal bold dotted line; violin representation. Mann-Whitney U test was performed to compare distribution in pre- and post-lockdown patients. Each dot (blue, pre-lockdown; red, post-lockdown) represents the values of a single patient.

© 2021 Thierry AR et al. *JAMA Network Open.*

eFigure 8. Comparison of the White Blood Cell Count in the Newly Diagnosed mCRC Patients From the Pre- and Post-Lockdown Study Cohorts (N=61)

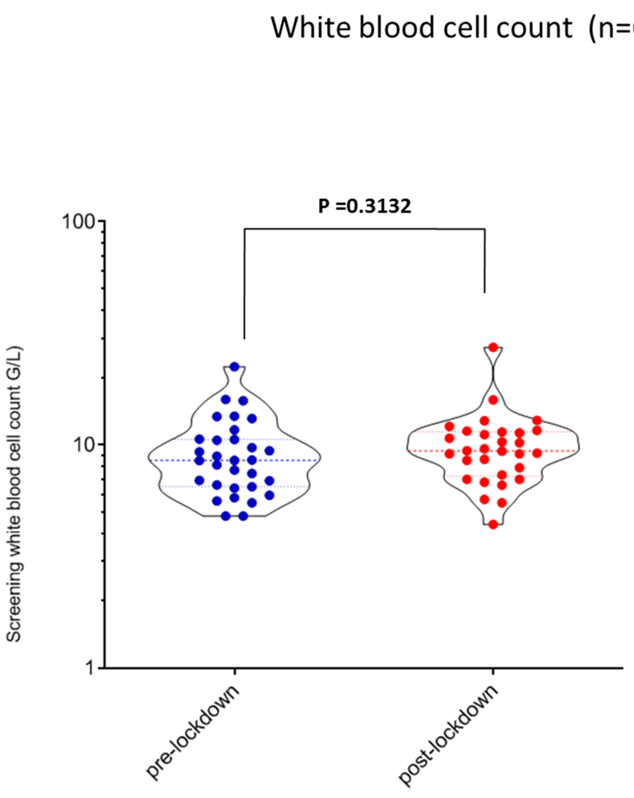

Table Analyzed	White blood cell count
Column B	pre-lockdown
vs.	vs,
Column A	post-ockdown
Mann Whitney test	
P value	0,3132
Exact or approximate P value?	Exact
P value summary	ns
Significantly different (P < 0.05)?	No
One- or two-tailed P value?	Two-tailed
Sum of ranks in column A,B	890,5 , 1001
Mann-Whitney U	394,5
Difference between medians	
Median of column A	8,510, n=31
Median of column B	9,385, n=30
Difference: Actual	0,8750
Difference: Hodges-Lehmann	0,8400

Group median is represented by a horizontal bold dotted line; violin representation. Mann-Whitney U test was performed to compare distribution in pre- and post-lockdown patients. Each dot (blue, pre-lockdown; red, post-lockdown) represents the values of a single patient.

© 2021 Thierry AR et al. *JAMA Network Open.*

eFigure 9. Comparison of the Carcinoembryonic Antigen (CEA) in the Newly Diagnosed mCRC Patients From the Pre- and Post-Lockdown Study Cohorts (N=61)

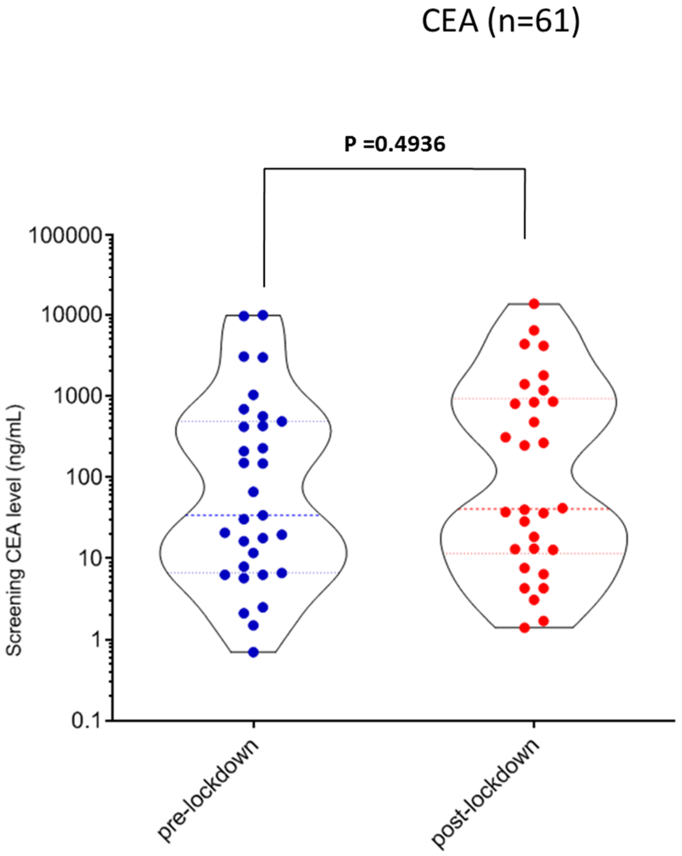

Table Analyzed	CEA Lockdown
Column B	pre-lockdown
vs.	vs,
Column A	post-lockdown
Mann Whitney test	
P value	0,4936
Exact or approximate P value?	Exact
P value summary	ns
Significantly different (P < 0.05)?	No
One- or two-tailed P value?	Two-tailed
Sum of ranks in column A,B	913 , 978
Mann-Whitney U	417
Difference between medians	
Median of column A	34,00, n=31
Median of column B	40,75, n=30
Difference: Actual	6,750
Difference: Hodges-Lehmann	6,700

Group median is represented by a horizontal bold dotted line; violin representation. Mann-Whitney U test was performed to compare distribution in pre- and post-lockdown patients. Each dot (blue, pre-lockdown; red, post-lockdown) represents the values of a single patient.

© 2021 Thierry AR et al. *JAMA Network Open.*

eFigure 10. Pearson r Correlation Analysis of the cirDNA, LDH, White Blood Cell Count, and CEA Levels in the Newly Diagnosed mCRC Patients From the Pre- and Post-Lockdown Study Cohorts

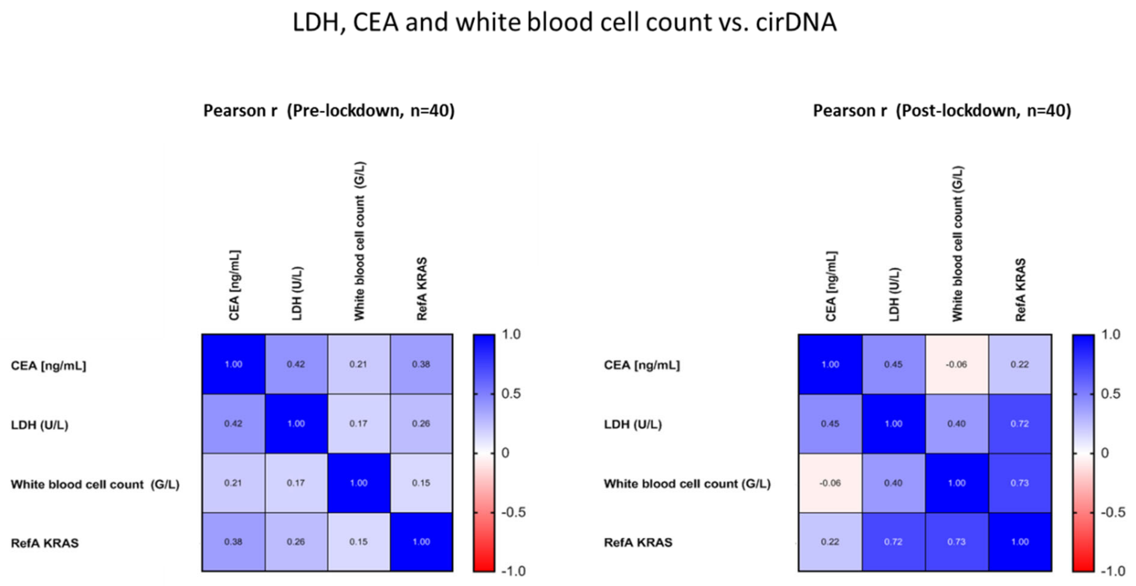

Sample size (n)				
	CEA [ng/mL]	LDH (U/L)	White blood cell count (G/L)	RefA KRAS
CEA [ng/mL]	31	29	31	31
LDH (U/L)	29	29	29	29
White blood cell count (G/L)	31	29	31	31
RefA KRAS	31	29	31	40

Sample size (n)				
	CEA [ng/mL]	LDH (U/L)	White blood cell count (G/L)	RefA KRAS
CEA [ng/mL]	30	27	30	30
LDH (U/L)	27	27	27	27
White blood cell count (G/L)	30	27	30	30
RefA KRAS	30	27	30	40

© 2021 Thierry AR et al. *JAMA Network Open.*

eFigure 11. Scatter Plots Showing the Correlation Between LDH and cirDNA Concentrations in the Pre- and Post-Lockdown Cohorts

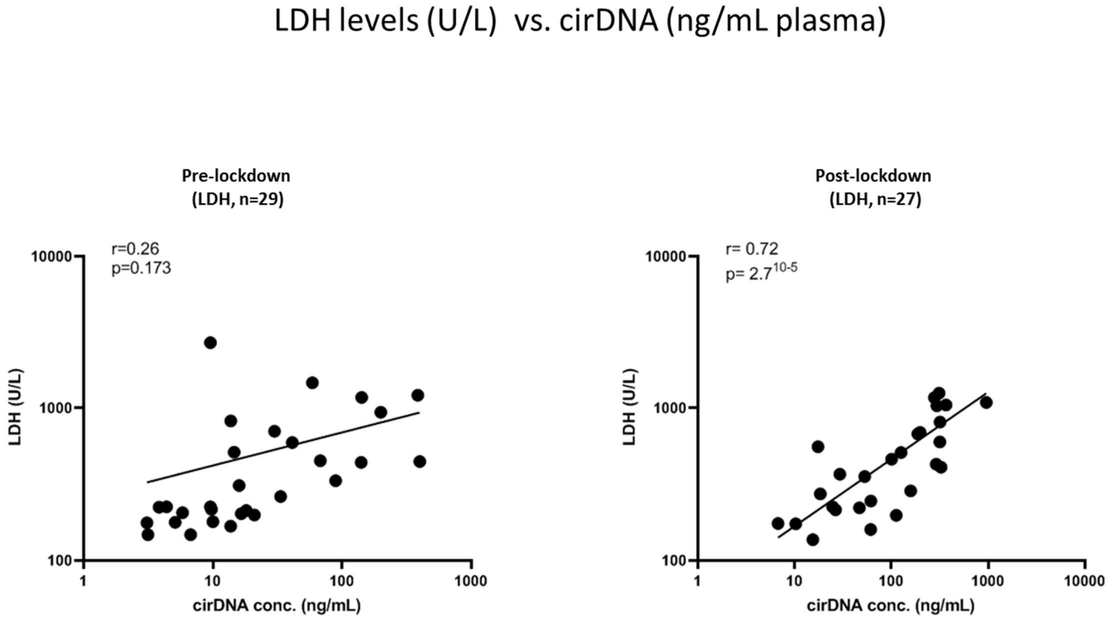

Each dot represents the values of a single patient; slanting lines are the regression lines as performed by the Spearman test.

© 2021 Thierry AR et al. *JAMA Network Open.*

eFigure 12. Scatter Plots Showing the Correlation Between White Blood Cell Count and cirDNA Concentrations in the Pre- and Post-Lockdown Cohorts

White blood cell count (G/L) vs. cirDNA (ng/mL plasma)

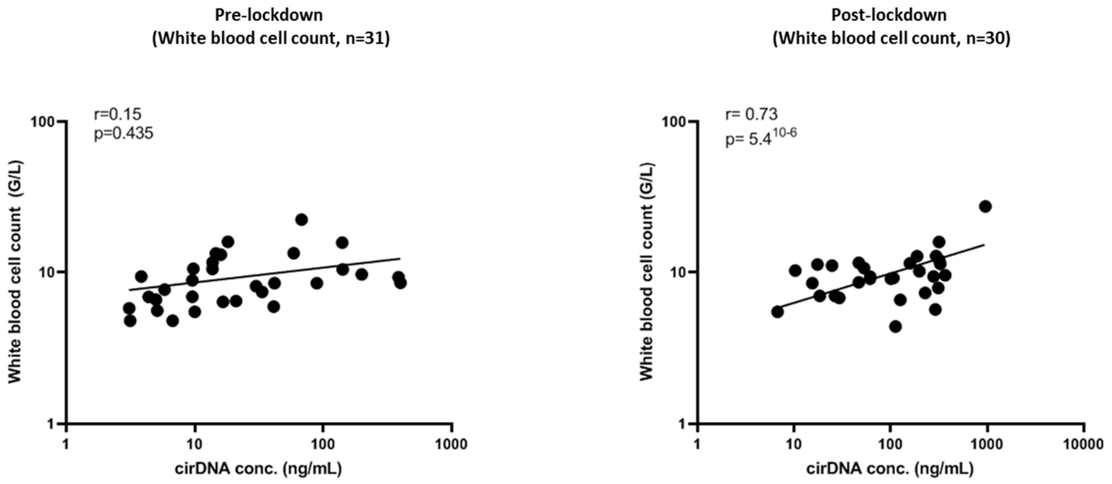

Each dot represents the values of a single patient; slanting lines are the regression lines as performed by the Spearman test.

© 2021 Thierry AR et al. *JAMA Network Open.*

eFigure 13. Scatter Plots Showing the Correlation Between CEA and cirDNA Concentrations in the Pre- and Post-Lockdown Cohorts

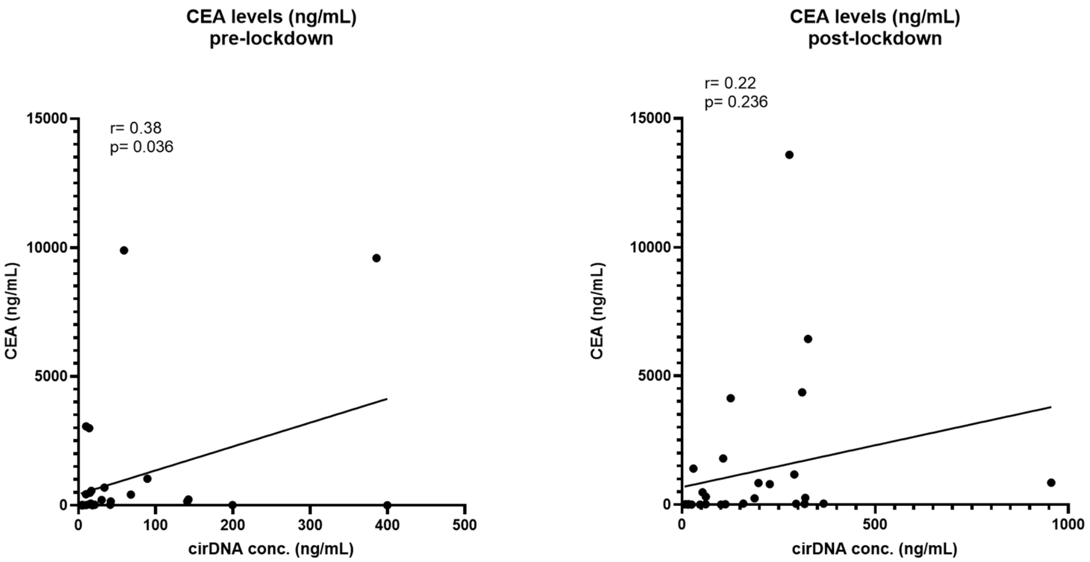

Each dot represents the values of a single patient; slanting lines are the regression lines as performed by the Spearman test.

eFigure 14. Scatter Plots Showing the Correlation Between White Blood Cell Count and LDH Concentration in the Pre- and Post-Lockdown Cohorts

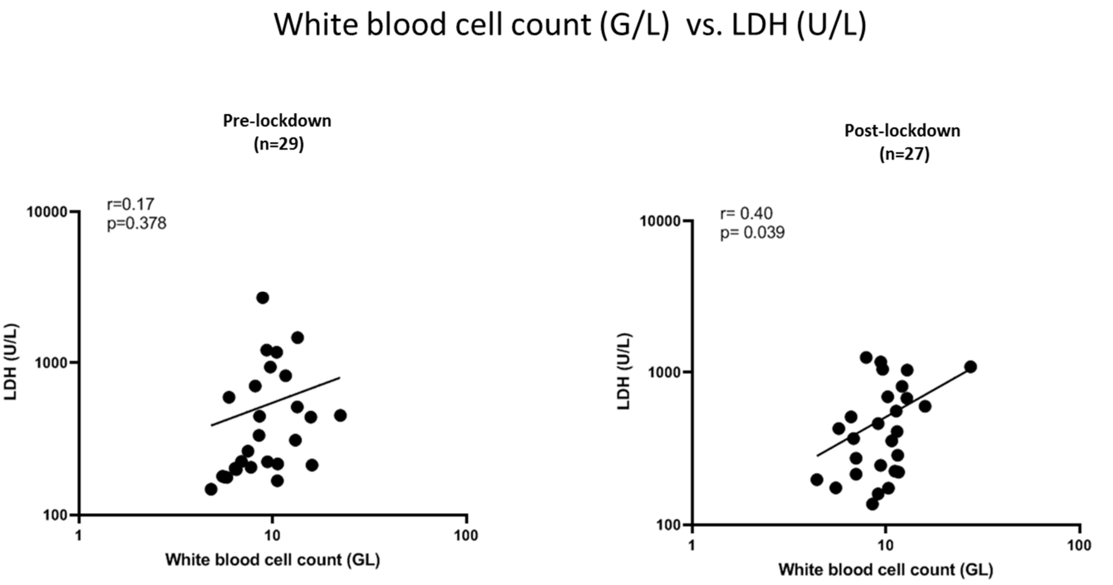

Each dot represents the values of a single patient; slanting lines are the regression lines as performed by the Spearman test.

© 2021 Thierry AR et al. *JAMA Network Open.*

eTable 1. CirDNA Analysis for Pre-Lockdown and Post-Lockdown Cohorts

**CirDNA analysis**	Cohorts N *(%)*			
	**Full study cohort**	**Pre-lockdown**	**Post-lockdown**	**Mann Whitney test pre- vs. post-lockdown (pvalue)**
**N**	80 ***(100%)***	40 ***(50%)***	40 ***(50%)***	
**CirDNA conc. (ng/mL plasma)**				
Median (IQR)	43,35 (15,47-195,4)	17,27 (9,57-399,7)	119,2 (43,38-315,8)	**p< 0.0001**
Min-Max	3,9-1852	3,09-396,6	6,74-1852	
**Mutational status**				
Wild type	32 ***(40%)***	18 ***(45%)***	14 ***(35%)***	
Mutant	48 ***(60%)***	22 ***(55%)***	26 ***(65%)***	
**Point mutation found in mutant patients**				
KRAS	**41**	**23**	**18**	
G12A	4	2	2	
G12V	15	8	7	
G12S	4	1	3	
G12D	5	2	3	
G12R	1	0	1	
G13D	6	5	1	
A146T	3	2	1	
A146V	1	1	0	
Q61H-AC	2	2	0	
BRAF				
V600E	5	3	2	
NRAS	**4**	**1**	**3**	
G12C	1	0	1	
G12D	1	1	0	
Q61R	1	0	1	
Q61H-AC	1	0	1	
**Mutant cirDNA concentration (ng/mL plasma)**				
Median (IQR)	5,26 (0,17-37,65)	1,99 (0,35-17,96)	14,21 (0,08-65,03)	**p= 0.5450**
Min-Max	0,014-510,5	0,014-510,5	0,02-401,9	
**Mutant allele frequency (%)**				
Median (IQR)	7,20 (0,64-21,63)	10,45 (0,88-19,22)	6,18 (0,45-21,96)	**p= 0.6932**
Min-Max	0,009-57-93	0,1-52,20	0,009-57,93	

© 2021 Thierry AR et al. *JAMA Network Open.*

eTable 2. Cox Models Data on Median Survival of mCRC Patients

	Dichotomisation: cirDNA conc. cut-off (ng/mL plasma)	effectif (n)	median survival (months)	median survival equal or below cut-off (months)	median survival over cut-off (months)	Hazard Ratio (HR)	CI95% of median survivals	Cox models (p value)
Figure 3A	NA	135	48,7				[13,6-20,6]	
Figure 3B	24,4	135	48,7	20	14,7	1,74	[1,2-2,6]	0,005
Figure 3C	100	135	48,7	19,3	8,8	2	[1,2-3,3]	0,009
legend:								
NA: Non Applicable								
CI95%: 95% Confidence Interval								

© 2021 Thierry AR et al. *JAMA Network Open.*
